# Encouraging physician appropriate prescribing of non-steroidal anti-inflammatory therapies: protocol of a randomized controlled trial [ISRCTN43532635]

**DOI:** 10.1186/1472-6963-4-21

**Published:** 2004-08-24

**Authors:** Malcolm Doupe, Alan Katz, Brent Kvern, Lori-Jean Manness, Colleen Metge, Glen TD Thomson, Laura Morrison, Kat Rother

**Affiliations:** 1 Primary Health Care Research Unit, St Boniface Research Centre, Winnipeg, Canada; 2 Department of Family Medicine, University of Manitoba, Winnipeg, Canada; 3 Department of Continuing Medical Education, University of Manitoba, Winnipeg, Canada; 4 Department of Patient Health, Merck Frosst Canada Ltd., Kirkland, Canada; 5 Faculty of Pharmacy, University of Manitoba, Winnipeg, Canada; 6 CIADS Research, Centre for Inflammatory and Arthritic Disease Studies, Winnipeg, Canada

## Abstract

**Background:**

Traditional non-steroidal anti-inflammatory drugs (NSAIDs) are a widely used class of therapy in the treatment of chronic pain and inflammation. The drugs are effective and can be relatively inexpensive thanks to available generic versions. Unfortunately the traditional NSAIDs are associated with gastrointestinal complications in a small proportion of patients, requiring costly co-therapy with gastro-protective agents. Recently, a new class of non-steroidal anti-inflammatory agents known as coxibs has become available, fashioned to be safer than the traditional NSAIDs but priced considerably higher than the traditional generics. To help physicians choose appropriately and cost-effectively from the expanded number of anti-inflammatory therapies, scientific bodies have issued clinical practice guidelines and third party payers have published restricted reimbursement policies. The objective of this study is to determine whether an educational intervention can prompt physicians to adjust their prescribing in accordance with these expert recommendations.

**Methods:**

This is an ongoing, randomized controlled trial. All primary care physicians in Manitoba, Canada have been randomly assigned to a control group or an intervention study group. The educational intervention being evaluated consists of an audit and feedback mechanism combined with optional participation in a Continuing Medical Education interactive workshop. The primary outcome of the study is the change, from pre-to post-intervention, in physicians' appropriate prescribing of non-steroidal anti-inflammatory therapies for patients requiring chronic treatment. Three classes of non-steroidal anti-inflammatory therapies have been identified: coxib therapy, traditional NSAID monotherapy, and traditional NSAID therapy combined with gastro-protective agents. Appropriate prescribing is defined based on international clinical practice guidelines and the provincial drug reimbursement policy in Manitoba.

## Background

Traditional non-steroidal anti-inflammatory drugs (NSAIDs) are a widely prescribed class of therapy used to relieve pain and inflammation. The drugs have been shown to be effective for a variety of common disorders (hence their widespread use), most notably chronic osteoarthritis and rheumatoid arthritis. They are relatively inexpensive due to the available generic versions, but unfortunately have clinically important drawbacks related to their gastrointestinal (GI) toxicity [1]. Each year, about 1% to 1.5% of patients taking traditional NSAIDs experience serious GI side effects such as perforations, ulcers, and bleeding [2-5]. When multiplied by the total number of NSAID users this translates into significant patient morbidity and mortality [1,6] and is associated with considerable health care costs related to hospitalizations or to the prescribing of expensive gastro-protective agents (GPAs) [7-12]. The cause of this GI toxicity is the "non-selective nature" of traditional NSAIDs that block both cyclo-oxygenase-2 (Cox-2), an enzyme involved in the production of inflammation and pain, and Cox-1, a related molecule that functions in GI tract mucosal protection and platelet function [13].

In the last five years, a new class of non-steroidal anti-inflammatory agents has become available to physicians, fashioned specifically to be safer than the traditional NSAIDs but priced at least two to three times higher than the generic versions of traditional NSAIDs [14]. The new drugs preferentially inhibit Cox-2 enzymes as compared to COX-1 molecules, and therefore have been christened "Cox-2 selective inhibitors" or "coxibs" for short. Large clinical trials comparing the use of coxibs to traditional NSAIDs have lent support to the concept that the new agents offer an improved GI safety profile while maintaining comparable analgesic efficacy in patients with chronic arthritis [3,4,15]. The trials, however, have also hinted that the improved GI safety may be compromised by concomitant use of low-dose aspirin [4] and may come at the expense of some cardiovascular safety, although these data remain controversial [16,17]. Regardless, the introduction of these new anti-inflammatory agents has prompted the question: when is it appropriate and cost-effective to prescribe coxibs versus traditional NSAID monotherapy or traditional NSAIDs in combination with GPAs?

To assist physicians in selecting from the different classes of anti-inflammatory agents, various scientific bodies have published clinical guidelines and third party payers have issued restricted reimbursement criteria [18-24]. These guidelines and reimbursement criteria in general propose that, for the treatment of chronic osteoarthritis and rheumatoid arthritis, coxibs should be used in lieu of traditional NSAID monotherapy when patients have an elevated risk for serious GI events. Traditional NSAID therapy in combination with certain GPAs (misoprostol or proton pump inhibitors) is also recommended as an alternative for most high-risk patients. High-risk patients are identified as individuals who have one or more of the following clinical characteristics: a history of peptic ulcer disease; advanced age (over 65 years); concomitant use of corticosteroids or anticoagulants; multiple comorbid conditions; or use of high doses or multiple NSAIDs [1,5,6,25,26]. These high-risk patients are considered to benefit from the improved GI safety of coxibs or from the GI protection afforded by GPAs. Furthermore, coxibs and GPA co-therapy have been shown to be cost-effective in such high-risk patients, as the increased cost of the drugs is partially offset by significant reductions in morbidity and mortality and related expenses [14,27-29].

With these guidelines and policies on prescribing in place, it is now important for physicians to adjust their prescribing practices accordingly. This is true both for rheumatology specialists and for physicians in the primary care setting where osteoarthritis patients are frequently managed. Unfortunately, experience and educational research show that simply making guidelines available does not elicit behavioural change from physicians [30-33]. In the case of anti-inflammatory drug prescribing, there is already evidence showing that despite the availability of guidelines and reimbursement policies, physicians' choices of drugs remain suboptimal both in terms of the inappropriate use of traditional NSAIDs and the non-cost-effective use of coxibs [34-38].

The current use of anti-inflammatory drugs suggests a need for strategies that will prompt physicians to change their prescribing practice in accordance with the expert recommendations. Preferably, strategies should be investigated that have proved successful at altering physician behaviour in other settings.

In this paper, we describe a randomized controlled study, which we are presently conducting, to evaluate the impact of an educational intervention on primary care physicians' prescribing of non-steroidal anti-inflammatory therapies. The study, being conducted in the province of Manitoba, Canada, tests the hypothesis that this intervention will significantly improve physician appropriate prescribing of anti-inflammatory drugs in compliance with international clinical practice guidelines and Manitoba's restricted drug reimbursement policy [18-22]. The intervention being tested is modelled on proven approaches to changing physician behaviour; it consists of an audit and feedback mechanism with optional participation in a Continuing Medical Education (CME) interactive workshop. This manuscript summarizes Phases II to V of a larger initiative entitled the Manitoba Appropriate Anti-Inflammatory Utilization Initiative (MAAUI). The study began in November 2000 and is currently in the data analysis stage. In this paper, we relate in detail the protocol of this randomized controlled study.

## Methods

### Study population

MAAUI is a province-wide population-based study. All primary care physicians (non-specialists) registered with the College of Physicians and Surgeons of Manitoba and practicing in Manitoba since July 1995 were eligible to enter MAAUI. The study excluded residents, new physician graduates, physicians registered as specialists, and physicians who started practicing in the province of Manitoba after July 1995. The eligible study population thus totalled approximately 884 physicians.

All eligible physicians were automatically entered into the MAAUI protocol. Physicians who were randomized to intervention group of the research were given the option to withdraw from the study. Six family physicians who agreed to act as facilitators for the MAAUI CME workshop were not included in the study population.

### Study design

**Figure 1:**
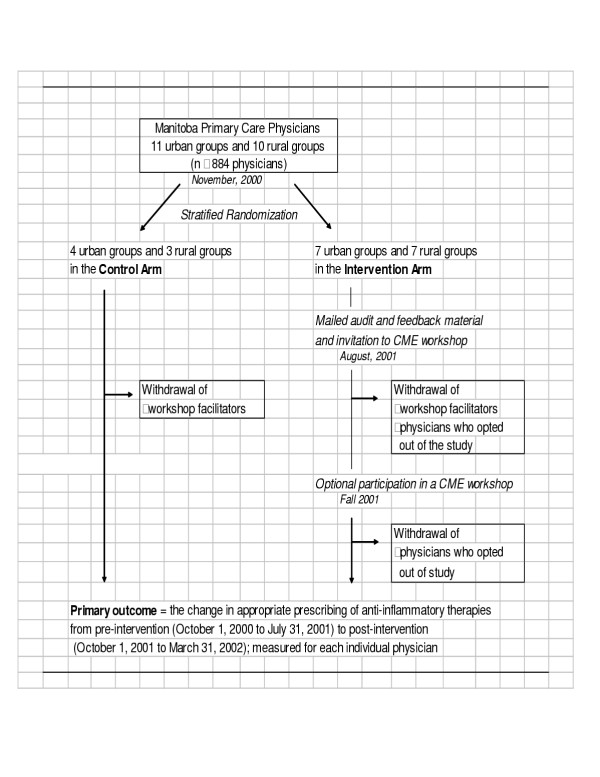
MAAUI study design

The MAAUI study design is depicted in Figure 1. Primary care physicians were allocated to the control and intervention arms of the study using a stratified randomization process. Specifically, study participants were divided into groups according to the geographical area of their practice within Manitoba. These groups were stratified by physicians' "urban" (within Winnipeg – the provincial capital, population size 676,700) or "rural" (outside of Winnipeg, population size 468,300) practice location. Randomization within each stratum was then carried out at the level of the group. The MAAUI research team originally identified 12 urban and 11 rural areas in Manitoba based on existing community boundaries within Winnipeg and the presence of distinct regional health districts outside of Winnipeg. Because one urban and rural area each contained very few primary care physicians (less than five), these areas were joined to neighbouring regions resulting in 11 urban and 10 rural groups. Urban and rural groups were randomized in such a way as to achieve a 1:2 ratio of control to intervention groups. Four of the urban groups and three of the rural groups were randomized to the control arm, and the remaining seven urban and seven rural groups were randomized to the intervention arm.

This study design was chosen for several reasons. First, it offered the scientific rigour that is associated with randomization and the use of a control group. It also ensured that both rural and urban Manitoban physician groups had an equal opportunity to take part in and learn from the study intervention. The study design was also particularly amenable to testing an intervention that required physicians to congregate in common locations (i.e. for the CME workshop). The 1:2 ratio of study groups helped to ensure that there were a sufficient number of study participants who would consider attending the CME workshops.

### The educational intervention

Physicians allocated to the intervention arm of MAAUI were mailed a package that included an introduction to the study, audit and feedback material, and an invitation to participate in a CME workshop. Physicians in the control arm received no package.

#### Audit and feedback material

**Figure 2:**
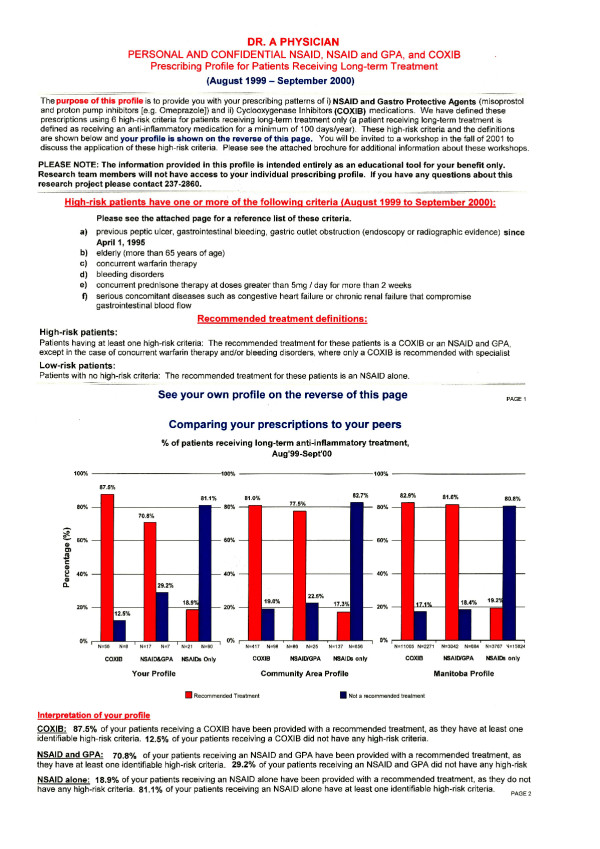
**Generic example of a Personalised Prescribing Profile. **The Personalised Prescribing Profile constituted the audit and feedback mechanism in MAAUI. Every physician in the intervention arm of the study was sent a Personalised Prescribing Profile by mail. a/ Side 1 of the profile provides a brief introduction to the purpose of the profile and lists the criteria for appropriate prescribing of coxibs, traditional NSAIDs (referred to as "NSAIDs" throughout the Profile), and traditional NSAID/GPA combination therapy. b/ On side 2, a bar chart illustrates for the physician his/her recent prescribing pattern of chronic non-steroidal anti-inflammatory therapies. The chart includes the number of patients to whom the physician has prescribed long-term treatment with each of the anti-inflammatory therapies between August 1999 and September 2000 and the proportion of these patients for whom the prescription was appropriate ("Recommended Treatment") or inappropriate ("Not a recommended treatment"). The chart also provides averaged statistics on appropriate and inappropriate prescribing for the physicians in the same geographical area and for primary care physicians in Manitoba overall.

The audit and feedback material consisted of a "Personalised Prescribing Profile" (see Figure 2). This profile illustrated for each physician his/her recent prescribing pattern of non-steroidal anti-inflammatory therapies and the appropriateness of this prescribing pattern in light of expert recommendations. Specifically, the profile contained a bar chart showing the number of patients to whom the physician had prescribed long-term treatment with coxibs, traditional NSAID monotherapy, or traditional NSAIDs in combination with GPAs between August 1999 and September 2000. The proportion of these patients with whom the prescription was appropriate was also included. Long-term treatment was defined as patients who received therapy for a minimum of 100 days during this period. The chart, in addition to showing the physician's own prescribing profile, also showed the average profile of primary care physicians in the same geographical area and the profile of Manitoban primary care physicians overall. The reverse side of the chart listed the criteria used to define appropriate prescribing. These criteria were based on international clinical practice guidelines and Manitoba's restricted drug reimbursement policy for coxibs, and were verified by a rheumatologist on the MAAUI research team [18-22]. The criteria identified coxibs or traditional NSAID/GPA combination therapy as the appropriate choice for patients at risk for serious GI complications. The criteria also identified coxibs as the only appropriate therapy for patients with bleeding disorders or patients taking concomitant anticoagulants (this latter recommendation was based in part on Manitoba's reimbursement policy for coxibs and in part on the Canadian Consensus guidelines, and reflected concerns over the anti-platelet effect of traditional NSAIDs [18,22]). The format of the Personalised Prescribing Profiles was developed by the MAAUI research team and was validated prior to the study intervention using two focus groups of family physicians.

The Personalized Prescribing Profiles were generated by Manitoba Health and the Manitoba Centre for Health Policy (MCHP) at the University of Manitoba, using administrative data from the Population Health Research Data Repository. This repository contains anonymized encounter-based records of individuals' interactions with the provincial health care system and is derived from information received by the Department of Health, Province of Manitoba, as part of the routine provision of health care in the province. The repository includes Physician Registry files, Medical Claims and Hospital discharge files, as well as Drug Programs Information Network (DPIN) files. The DPIN contains records of all drugs dispensed by Manitoba pharmacies, regardless of who is responsible for payment.

Because of the sensitive nature of the Personalised Prescribing Profiles, the individualized profiles were not seen by the MAAUI research team. Instead the packages containing the profiles were assembled and mailed out by Manitoba Health with the assistance of an independent researcher, hired by the MAAUI research team, who signed a confidentiality agreement with Manitoba Health.

#### CME workshop

The CME workshop for MAAUI was entitled "The Utilization and Prescribing of Anti-inflammatory Drugs in Osteoarthritis". The workshop focused specifically on the prescribing for osteoarthritis, as it was felt that people with this disease account for a large proportion of the chronic non-steroidal anti-inflammatory use by primary care practices [39].

Eleven CME workshops were held throughout Manitoba (four within Winnipeg and seven outside of Winnipeg) and physicians in the intervention group were invited to voluntarily attend one of the 11. The workshops were free of charge and were approved for 3.0 hours of MAINPRO-M1 credits (continuing education credits awarded by the College of Family Physicians of Canada for group learning activities). Each workshop was facilitated by a trained family physician.

The workshops included three components: an introductory video, a decision tree, and a case study portion. The introductory video provided the history and rationale of MAAUI, explained aspects of the Personalised Prescribing Profile, and introduced the decision tree. The decision tree depicted a stepwise approach to the diagnosis and treatment of osteoarthritis. This aid was designed by a rheumatologist on the MAAUI research team specifically for use in the study and was based on international clinical practice guidelines and Manitoba's drug reimbursement policy [18-22]. A take-home copy of the decision tree was provided to each workshop participant (see Additional file 1). Finally, the case study portion of the workshop consisted of studies exploring both the diagnosis and appropriate treatment of osteoarthritis. These case studies were supplied from an existing CME curriculum entitled "Clinical Scenarios in Osteoarthritis" [40].

The overall MAAUI educational intervention, including both the audit and feedback material and the optional CME interactive workshop, was chosen by the MAAUI research team based on the success of similar interventions in the past. Audit and feedback mechanisms have shown moderate success in changing physician behaviour in past studies [41]. Equally, the addition of a continuing education workshop to an existing intervention has been shown to increase the impact on physician behaviour [42]. Workshops with interactive elements, such as case studies, have also been found to be more successful at influencing physician behaviour than purely didactic lectures [43]. Affordability, practicality, and reproducibility were also considered when designing the intervention.

### Outcomes

#### Principal outcomes

The primary goal of MAAUI is to evaluate the impact of the educational intervention on physician appropriate prescribing of non-steroidal anti-inflammatory therapies for patients requiring long-term treatment. To this end, four principal outcome measures have been developed:

• the change in appropriate prescribing of all non-steroidal anti-inflammatory therapy (including coxibs, traditional NSAIDs, and traditional NSAID/GPA combination therapy) from pre-to post-intervention;

• the change in appropriate prescribing of coxibs from pre-to post-intervention;

• the change in appropriate prescribing of traditional NSAID/GPA combination therapy from pre-to post-intervention; and

• the change in appropriate prescribing of traditional NSAID monotherapy from pre-to post-intervention.

These primary outcomes will be evaluated at the level of the individual physician and will be calculated using administrative data similar to that used to create the Personalised Prescribing Profiles. The change in appropriate prescribing (AP) for a given physician will be calculated by the physician's rate of appropriate prescribing during a 6-month period post-intervention (October 1^st ^2001 to March 31^st ^2002) minus this rate during a 10-month period immediately pre-intervention (October 1^st^, 2000 to July 31^st^, 2001). The rate of appropriate prescribing will be defined as the number of patients prescribed long-term treatment in whom the treatment was appropriate, divided by the total number of patients prescribed long-term treatment, multiplied by 100%. Otherwise expressed:

Physician's ΔAP = Post-intervention AP - Pre-intervention AP



Appropriateness of prescribing will be calculated by a researcher who is blinded to participants' study-arm allocation, and will be based on the same criteria as listed in the Personalised Prescribing Profiles (see Figure 2a). In the event that a physician has fewer than five patients on long-term treatment in either the pre- or post-intervention period, that physician's data will be withheld from analysis to ensure confidentiality of the patients.

#### Secondary outcomes

In addition to the four principal outcomes, the MAAUI study has a number of prospectively-defined secondary outcomes:

• The study will re-evaluate the primary outcomes in the following two physician subgroups: physicians who received the audit and feedback material and attended the CME workshop; physicians who received the audit and feedback material and chose not to attend the CME workshop. This analysis will help to determine the impact that the different components of the educational intervention had on physician behaviour.

Specifically in the subgroup of physicians who attended the CME workshop:

• The study will measure the change in physician knowledge of osteoarthritis from pre-CME workshop to immediately post-CME workshop and the change in knowledge from pre-CME workshop to five months post-CME workshop. These two outcomes should provide insight into physicians' retention of information following a CME workshop and will allow us to examine any relationship between change in physician knowledge and change in physician prescribing behaviour.

• The study will also survey physicians' perceived change in prescribing behaviour at five months post-intervention. This outcome, compared with the principal study outcomes, will provide insight into the accuracy with which physicians' discern their own prescribing practices.

• The study will collect process-related measures in order to mark areas for improvement in the study interventions. Specifically, physicians' impressions of the Personalised Prescribing Profile and CME workshop will be surveyed using the questionnaire distributed immediately following the CME workshop and using focus-group discussions at the end of the study follow-up. All workshop participants will be invited to attend a follow-up focus group (one for urban participants and one for rural participants). A separate focus group will be held for the workshop facilitators.

### Baseline data collection

The following data were collected at baseline (October 1^st^, 2000 to July 31^st^, 2001) as control measurements: i) physician demographics including sex, urban or rural practice location, and the number of years in practice; ii) physician volume of non-steroidal anti-inflammatory therapies prescribed annually for patients requiring chronic therapy; and iii) physician rate of appropriate prescribing of NSAIDs. These latter two measures were assessed for each of coxibs, traditional NSAID/GPA combination therapy, and traditional NSAID monotherapy.

### Sample size and statistics

Sample size was calculated based on the need to detect at least a 10% improvement in physician practice patterns associated with the intervention, for clinical significance. Alpha error was set at 1% because of the multiple primary outcomes and power was set at 80%. It was also assumed in the sample size calculation that physician baseline demographic measures and baseline practice patterns would contribute an additional 10% variation to the outcomes. Based on these figures and using sample size techniques for multivariate regression analysis [44], we estimated the need for a minimum of 116 physicians in the control group and intervention group respectively.

Multivariate regression analysis will be used to determine if the study intervention has a significant impact on the primary outcomes. Baseline data will be included as control measures and interaction effects between these baseline data and the intervention will also be evaluated.

### Ethics

The MAAUI protocol has been approved by the Health Research Ethics Board, Faculty of Medicine, University of Manitoba, and by the Health Information Privacy Committee of Manitoba Health which is responsible for approving research projects that use personal health information held by a government department.

## Competing interests

The MAAUI study is funded by Merck Frosst Canada Ltd., a pharmaceutical company that produces a range of non-steroidal anti-inflammatory therapies including a coxib and various traditional NSAIDs. Merck Frosst's involvement in the study is limited to representation (by L-JM) on the steering committee, which is the governing body responsible for approving all aspects of study development and implementation. Merck Frosst will not participate in data analysis and will not have access to confidential individual patient or physician data.

AK, CM, and GT have received speaking fees from Merck Frosst Canada. KR is the scientific writer for this manuscript, employed by the PHCRU.

All of these competing interests were not specifically disclosed to study participants. Merck Frosst's sponsorship of the study, however, was disclosed in the consent form signed by participants at the MAAUI CME workshops.

## Authors' contributions

MD, AK, BK, and CM conceived of the study. MD, AK, L-JM, CM, and GT contributed to the development of the protocol. GT created the decision tree and list of criteria for appropriate prescribing. GT also chaired the MAAUI project steering committee. LM is project managing the study. KR performed the literature review for this manuscript and wrote the first draft and coordinated all subsequent revisions. All authors approved the final manuscript.

## Pre-publication history

The pre-publication history for this paper can be accessed here:



## Supplementary Material

Additional file 1 **Decision Tree: take-home copy**The decision tree was a component of the MAAUI CME workshop. Physicians were introduced to the decision tree during the workshop and were provided with a two-sided take-home copy. The decision tree depicts a stepwise approach to the diagnosis and treatment of osteoarthritis. a/ Side 1. b/ Side 2. Of note, on side 2 of the decision tree the term "NSAID" denotes traditional NSAIDs. click here for file
