# Supplementary material for: Encouraging physician appropriate prescribing of non-steroidal anti-inflammatory therapies: protocol of a randomized controlled trial [ISRCTN43532635]
Source: BMC Health Serv Res. 2004 Aug 24;4:21. doi: 10.1186/1472-6963-4-21 (PMC516782; doi:10.1186/1472-6963-4-21)
Supplement: Additional file 1 — Decision Tree: take-home copyThe decision tree was a component of the MAAUI CME workshop. Physicians were introduced to the decision tree during the workshop and were provided with a two-sided take-home copy. The decision tree depicts a stepwise approach to the diagnosis and treatment of osteoarthritis. a/ Side 1. b/ Side 2. Of note, on side 2 of the decision tree the term "NSAID" denotes traditional NSAIDs. [file 1472-6963-4-21-S1.pdf]

University of Manitoba | Continuing Medical Education

Primary Health Care Research Unit

Approach for treatment of Osteoarthritis

DIAGNOSIS

↓

Osteoarthritis

Osteoarthritis Pain = **Joint Pain** + **Soft Tissue Pain** + **Other Factors**

Joint Pain
- bone pain
- cartilage pain
- mild inflammation

Soft Tissue Pain
- ligamentous strain
- tendonitis
- bursitis

Other Factors
- excessive demand on joints (work, weight etc.)
- disrupted sleep

TREATMENT

Mechanical

= reduce excessive demand on joints and soft tissues:

- weight loss
- rest
- orthotics and footwear
- other devices
  - splints and braces
  - canes and walkers

Physical

= reduce pain with physical measures:

- applications of cold and heat
- massage
- acupuncture
- TENS

= appropriate stretching and strength exercises

= aerobic conditioning

Pharmacological

- acetaminophen to maximum dose (4 gms/day)
- topical NSAID for small joints
- intra-articular hyaluronic acid injections
- injection of bursitis with corticosteroids
- pain relief/sedation for disturbed sleep

← Consult Physiotherapy and Occupational therapy →

Anti-inflammatory Therapy for osteoarthritis

Please turn page over

Sponsored by:
MERCK FROSST
Discovering today for a better tomorrow.

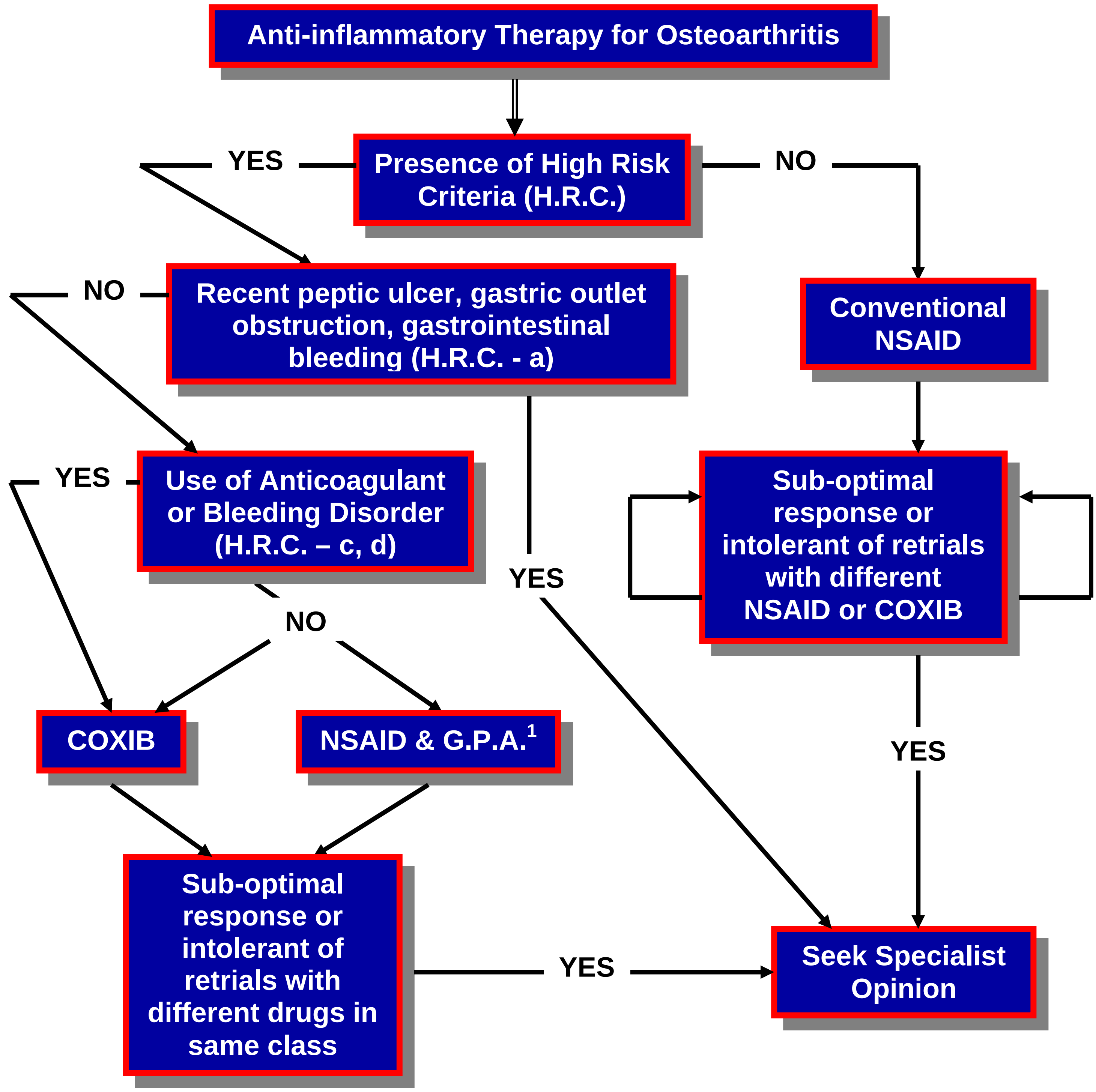

High Risk Criteria (H.R.C.)

a) previous peptic ulcer, gastric outlet obstruction, gastrointestinal bleeding
b) elderly (more than 65 years of age)
c) concurrent warfarin therapy
d) bleeding disorders
e) concurrent prednisone therapy at doses greater than 5mg / day for more than 2 weeks
f) serious concomitant diseases such as congestive heart failure or chronic renal failure that compromise gastrointestinal blood flow

[1] G.P.A. – gastro-protective agent
